# Prevalence and associated factors of wheezing illnesses of children aged three to five years living in under-served settlements of the Colombo Municipal Council in Sri Lanka: a cross-sectional study

**DOI:** 10.1186/s12889-018-5043-3

**Published:** 2018-01-11

**Authors:** Ruwanika Seneviratne, Nalika S. Gunawardena

**Affiliations:** 1 Ministry of Health, Nutrition and Indigenous Medicine, “Suwasiripaya”, No 385, Rev. Baddegama Wimalawansa Thero Mawatha, Colombo, 10 Sri Lanka; 2 World Health Organization Country Office for Sri Lanka, No 5, Anderson Road, Colombo, 05 Sri Lanka

**Keywords:** Ever-wheezing illnesses, Current-wheezing illness, Preschool child

## Abstract

**Background:**

A rising trend in Sri Lanka for asthma and wheezing illness is observed with higher morbidity in younger children and a paucity of related research. ‘Under-served settlements’ (USS) of Colombo Municipal Council (CMC) have poor living environments conducive to childhood wheezing. The objective was to describe the prevalence and associated factors of wheezing illnesses of three to five year old children living in low-income settlements in CMC.

**Methods:**

A cross-sectional study was conducted on 460 three to five year old children and their caregivers using cluster sampling among residents of two randomly selected USSs of CMC. An interviewer-administered questionnaire, observation checklist and data extraction form were used in data collection. A physician’s diagnosis of wheezing/whistling of the chest in their lifetime and a physician’s diagnosis of wheezing/whistling within the past twelve months were considered as ‘ever-wheezing illness’ and ‘current-wheezing illness’ respectively.

**Results:**

Mean age was 3.98 years (SD = ±0.64 years). A majority were males (51.3%) and Tamils (39.8%). Prevalence of ‘ever wheezing illness’ and ‘current wheezing illness’ were 38% (95% confidence interval (CI); 33.6%–42.5%) and 21.3% (95%CI; 17.6%–25.0%), respectively.

Maternal (*p* < 0.001) and paternal (*p* < 0.001) histories of wheezing, playing with soft toys in the sleeping area (*p* = 0.004), place of cooking combined with the living area (*p* = 0.03), unsatisfactory ventilation in the sleeping area (*p* < 0.001) were found to be significantly associated with increased ‘current wheezing’ through multivariate analysis in this study. Use of formula milk before six months of age (*p* = 0.014) was found to be protective through multivariate analysis.

**Conclusions:**

The magnitude of wheezing illnesses among three to five year old children residing in urban low-income settlements was found to be high. Children with a history of maternal and/or paternal wheezing should be targeted for early interventions to prevent wheezing illnesses. Interventions to avoid exacerbations should focus on the indoor environmental factors that were found to be associated with wheezing illnesses.

## Background

The global prevalence differences of asthma and wheezing are diminishing. As there is an observed increase in prevalence in areas where prevalence was previously low, the global burden of asthma and wheezing, however, continues to increase [1–3]; with the most significant increase noted among children between one to five years [4, 5]. Due to difficulties in demonstrating airflow limitation [6, 7] in young children, it is recommended to avoid the term ‘asthma’ in preschoolers with wheezing and airway inflammatory reactions and to use the term ‘wheezing illnesses’ instead [6, 8]. Wheezing illnesses affect the health, wellbeing and quality of life of the affected, impose a substantial burden on the family and the healthcare system, and can be particularly troublesome to diagnose and treat, especially when occurring in younger children [9–12]. As a result they have poorer symptom control and lower quality of life than older children with wheezing [9, 13]. Research evidence on prevalence of wheezing illnesses and associated host or genetic and environmental factors among children of preschool age is sparse in low and low-middle income countries [14, 15].

In Sri Lanka in 2012, diseases of the respiratory system were the third leading cause of hospitalization and accounted for 14.4% of deaths [16] among 1,750,912 one to five year olds in the country, which comprises 8.6% of the total population [17]. According to the Global Burden of Disease study, in 1990, asthma led to 33,000 total years of life lost (YLLs) in the country (involving 1% of the total population) and was ranked as the 23rd cause of YLLs in the country. With a staggering 75% increase, the rank for asthma rose to the 11th position in 2010, with a total of YLLs increasing to 57,000 involving 1.8% of the total population [18]. In Sri Lankan studies, the prevalence and factors associated with asthma and wheezing illnesses have mainly been conducted among children of five to 14 years age group [19–22]. Though statistics indicate a rising trend, there is a dearth of surveys in the younger preschool child.

The Colombo Municipal Council (CMC) is a densely populated area of 37.3 km^2^ in a rapidly urbanized location. The estimated resident population is 665,000 [17] and a ‘floating population’ of 400,000–500,000 commute daily for employment and education purposes (Colombo Municipal Council Data, 2012, unpublished). The CMC also has the largest concentration of poor [23] living in designated under-served settlements (USS) situated in and around open sewers, waste disposal sites, and in proximity to industrial plants with toxic emissions and polluted environments [24]. In addition, many social problems such as high levels of alcohol and drug abuse, high youth unemployment rates, high levels of crime and violence, low levels of education and, marital instability, are also common [23, 24]. Up to date no surveys have been carried out in underserved communities to assess its burden of childhood asthma, wheezing illnesses or associated factors, particularly in children of the preschool age.

In this background, the objective of this article is to present the burden of wheezing illnesses and its associated factors among children aged three to five years living in urban low-income conditions in Colombo, the commercial capital of Sri Lanka. As the age category of three to five years and geographically demarcated USS are areas which have hitherto not received much attention of research, this study was carried out to generate new information.

## Methods

This was a descriptive cross-sectional study carried out in the USSs of the CMC from May 2015 to December 2015.

The study populations were children aged three to five years living in households in USS and their caregivers. A child-caregiver pair living in a household in the USS of the CMC where the child was aged three to five years and where the caregiver had been living with the child for one year or more was included. A caregiver was defined as an adult living in the same household who identified herself or himself as the person responsible for the care of that child. Child-caregiver pairs in which the child did not possess the Child Health Development Record (CHDR) and in which the caregivers could not be contacted to be interviewed even after three attempts during the period of data collection were excluded from the study.

The estimated sample size was 460. In the absence of estimates of prevalence of wheezing in children aged three to five years in Sri Lanka, the sample size was calculated based on 34% global prevalence of wheezing in children aged one to five years [25, 26] using an absolute precision of 5%, a design effect of 1.2 based on literature with a 10% non-response rate. The CMC is subdivided into six ‘districts’ which are further divided into 47 municipal ‘wards’. This study was carried out in two randomly selected ‘districts’ and in all of the 20 municipal ‘wards’ of the selected two districts. The study units were selected using cluster sampling method. Each municipal ‘ward’ formed a cluster and thus there were 20 clusters requiring 23 study units to be drawn from each cluster. Within each cluster, the starting point for the cluster survey was selected by dropping a pin on a detailed road map showing the underserved settlements and selecting the road on which the pin head fell. From the first household on the right hand side of the randomly selected road 23 study units were recruited continuously until the required cluster size was obtained. Only one eligible child-caregiver pair per household was selected and, in houses where there was more than one, random selection was carried out.

Three study instruments were used to collect data in the study: a structured, pre-tested, interviewer-administered questionnaire, a pre-tested observation checklist to facilitate assessment of the indoor and outdoor environment and a data extraction form to note the birth weight and immunization status from the CHDRs of the child selected for the study.

Two pre-intern doctors with previous experience in data collection in the field were recruited for data collection purposes. They were trained on: the objectives of the study, obtaining informed consent from the participants, administering the questionnaire and other study instruments with minimal disturbance of day-to-day activities and on obtaining reliable and accurate data.

The operational definitions of ‘ever-wheezing illness’ and ‘current-wheezing illness’ used in the survey were, a physician’s diagnosis of wheezing/whistling of the chest in their lifetime and a physician’s diagnosis of wheezing/whistling within the past twelve months, respectively.

In operationalizing the variables on potential factors associated with wheezing illness, ‘low birth weight' was defined as a birth weight of less than 2500g. Liquid Petroleum Gas (LPG) was considered as ‘clean’ fuel while use of firewood and kerosene were amalgamated together as ‘unclean’ type of cooking fuel. ‘Unsatisfactory ventilation’ was considered if there were no openings or having small openings the size of a lid of a standard bottle of jam to the size of a standard brick, in the cooking and sleeping areas. Ventilation was considered as ‘satisfactory’ if there were windows or exhaust fans. ‘Exposure to pets’ was defined if there had been cats, dogs or birds in the household for more than twelve months prior to the study. ‘Tendency for mould formation’ was defined as ‘higher tendency’ if there was damp walls, leaking roof or both in the household while lower tendency if there were no problems. Either daily or at least four times a week usage of mosquito coils was defined as ‘exposure to irritant smoke from mosquito coils’. Use less than four times a week or occasionally used was considered as less exposed. ‘Exposure to vehicular emissions’ was considered if the house was located either on a main road, or near a by-road with heavy or infrequent traffic. If the house was located in an enclosed locality called “watta” or ‘garden’ with an access road with no traffic, it was considered less exposed. ‘Exposure to emissions from activities/industries was defined as positive if there were places of work/activities/industry generating emissions located within 150 m from the household.

To find out the history of presence of ‘severe’ wheezing symptom episodes the following question was included in the questionnaire: ‘has this child ever developed severe symptoms- with marked difficulty in breathing and audible wheezing/ whistling of the chest?’ For information on episodes with ‘mild’ symptoms the following question was included in the questionnaire: ‘has this child ever developed mild symptoms- with mild difficulty in breathing with or without wheezing/whistling of the chest?’

Prevalence estimates were also estimated for ‘ever wheezing illness’ and ‘current wheezing illness’ among children aged three to five years according to age and sex distribution of the children in the study.

All the prevalence estimates are presented along with 95% confidence intervals (CI). The potential factors associated with ‘current wheezing illness’ were assessed in bivariate analysis using prevalence odds ratio (OR) along with 95% CI and chi square (χ^2^) test at 5% to assess the significance. All variables that were assessed with bivariate analysis have been adjusted for in the regression model to identify unconfounded factors associated with ‘current wheezing illness’. Analyses were done by using Statistical Package for Social Sciences (SPSS) version 22.

## Results

### Description of the study units

All the child-caregiver units invited, participated in the study with a response rate of 100% (460/460). In all the households identified following the application of the described sampling technique (*n* = 460, 100%) the caregivers were contacted within 3 attempts. The mean age of the children was 3.98 years (SD = ±0.64 years). For 90% of children (*n* = 414), the caregiver was the mother. The caregiver was the grandmother for 6.3% (*n* = 29), was an aunt for 3.1% (*n* = 14) and a non-relative female from the neighbourhood in 0.6%.

In the study approximately half (51.5%) were in the age category of four to five years, males (51.3%) and attended preschool (57%). According to the CHDRs of the children in this study, only 6.7% (*n* = 31) had an incomplete immunization status. The sample comprised approximately one third of Sri Lankan Tamils (39.8%), Muslims (30.6%), and Sinhalese (27.4%) and a majority had siblings (74.3%, *n* = 342) (Table 1).

**Table 1:** Distribution of children by socio demographic characteristics

Characteristic	Number (*N* = 460)	Percentage (%)
Age (in completed years)		
3 up to 4 years	223	48.5
4 up to 5 years	237	51.5
Sex		
Male	236	51.3
Female	224	48.7
Ethnicity		
Sri Lankan Tamil	183	39.8
Sinhalese	126	27.4
Muslim	141	30.6
Indian Tamil	5	1.1
Burgher	5	1.1
Having siblings		
Yes	342	74.3
No	118	25.7
Attended preschool		
Yes	262	57.0
No	198	43.0

A majority of the mothers were housewives (84.8%, *n* = 390) and a majority of the fathers were employed (98.5%, *n* = 453). Nearly 59% of the mothers and 55% of the fathers had been educated only up to grades nine to eleven at school. Approximately 5.9% of fathers and 2.6% of mothers had not received schooling. The median family monthly income was Rupees 28,000 (USD 193.1) with an interquartile range of Rupees 20,000–35,000 (USD 137.9–241.3). The main type of fuel used was Liquid Petroleum Gas (LPG) (62.2%, *n* = 286). Kerosene was used by 35.6% (*n* = 164) and wood by 2.2% (*n* = 10) of the households as cooking fuel. None of the households used electricity for cooking.

### Prevalence of wheezing illnesses

The prevalence of ‘ever wheezing illness’ and ‘current wheezing illness’ within past twelve months duration among three to five year of children living in underserved settlements of the CMC were 38% (95% CI; 33.6–42.5) and 21.3% (95%CI; 17.6–25.0), respectively.

The prevalence of ‘ever wheezing illness’ and the prevalence of ‘current wheezing illness’ among males and females showed no significant difference in this sample. Of the children who had ‘ever wheezing illness’, 73.1% (*n* = 128) had documented evidence of a diagnosis (Table 2).

**Table 2:** Prevalence estimates of ‘ever wheezing illness’ and ‘current wheezing illness’ by sex

Description	Number	Prevalence	95% CI*
Ever wheezing	175	38.0	33.6–42.5
Sex			
Male (*n* = 236)	96	40.7	34.4–46.9
Female (*n* = 224)	79	35.3	29.0–41.5
Current wheezing	98	21.3	17.6–25.0
Sex			
Male (*n* = 236)	47	19.9	14.8–25.0
Female (*n* = 224)	51	22.8	17.3–28.3

*CI Confidence Intervals

Of children with a history of ‘ever wheezing illness’, the mean number of episodes of wheezing experienced during the life time was 6.4 episodes (SD = ±7.03, minimum-2 and maximum-30). All the children with ‘ever wheezing illnesses’ had experienced episodes of ‘severe’ symptoms (*n* = 175, 100%) and episodes of ‘mild’ symptoms (*n* = 175, 100%). For severe wheezing illness (76%, *n* = 133) and mild wheezing illness (44%, *n* = 77), most of the caregivers had sought treatment from the state sector. For the last episode of wheezing illness, a majority of caregivers had accessed healthcare from state sector hospitals (61.7%, *n* = 108) and had been treated at the out-patient department (92%, *n* = 161). A great majority has been both nebulised and given oral treatment (90.9%, *n* = 159). None of the caregivers (*n* = 0, 0%) had sought indigenous medicine for treatment of wheezing episodes. Only 13.0% (*n* = 60) and 8.5% (*n* = 39) of children had a history of food allergy and eczema respectively.

### Factors associated with ‘current wheezing’ illnesses

Results of the analyses conducted for all the factors associated with ‘current wheezing’ are shown in Table 3. A logistic regression model was developed for assessing factors associated with ‘current wheezing’, after adjusting for confounders. All factors that were assessed with bivariate analysis were included in the regression model.

**Table 3:** Factors associated with wheezing – from bivariate and multivariate analyses

	Variables	*N*	Unadjusted OR* (95% CI**)	*p* value	Adjusted OR (95% CI)	*p* value
1	Age 3 up to 4 years	383	0.88 (0.55–1.39)	0.59	0.74 (0.34–1.58)	0.43
2	Male sex	383	1.05 (0.64–1.61)	0.84	1.88 (0.98–3.58)	0.056
3	Low birth weight	383	1.01 (0.53–1.92)	0.96	1.62 (0.66–3.95)	0.29
4	Not exclusively breast fed	383	0.34 (0.05–2.44)	0.27	11.05 (0.89–137.21)	0.06
5	Introduced to formula milk before 6 months	301	0.54 (0.32–0.92)	*0.022*	0.39 (0.19–0.83)	*0.014*
6	Ethnicity- Sinhalese	383	0.96 (0.57–1.61)	0.87	0.67 (0.31–1.46)	0.32
7	Having siblings	383	0.93 (0.55–1.58)	0.79	0.88 (0.42–1.87)	0.75
8	Attending preschool	383	0.83 (0.52–1.31)	0.43	1.09 (0.50–2.38)	0.82
9	Food allergy present	383	1.44 (0.75–2.74)	0.26	2.54 (0.92–7.02)	0.07
10	Having eczema	383	1.32 (0.55–3.13)	0.53	0.39 (0.09–1.67)	0.21
11	Food allergy in family	383	1.83 (1.01–3.31)	*0.044*	1.43 (0.54–3.78)	0.47
12	Eczema in family	383	2.35 (0.85–6.51)	0.08	4.07 (0.87–18.96)	0.07
13	Maternal asthma	383	8.88 (5.26–14.99)	*p < 0.001*	11.21 (5.64–22.29)	*p < 0.001*
14	Paternal asthma	383	6.58 (3.69–11.73)	*p < 0.001*	7.98 (3.64–17.49)	*p < 0.001*
15	Pets at home	383	1.56 (0.85–2.86)	0.14	0.98 (0.43–2.25)	0.97
16	Place of sleep (hardboard/bare floor/mat)	383	1.64 (1.005–2.688)	*0.047*	1.17 (0.57–2.42)	0.66
17	Unsatisfactory ventilation in sleeping area	383	2.42 (1.49–3.85)	*p < 0.001*	4.38 (2.16–8.89)	*p < 0.001*
18	Use of bed nets	383	1.71 (1.008–2.904)	*0.045*	1.25 (0.58–2.65)	0.57
19	Play with soft toys	383	2.14 (1.33–3.43)	*0.001*	2.65 (1.35–5.18)	*0.004*
20	Tendency of mould formation	383	2.20 (1.37–3.52)	*0.001*	1.54 (0.79–2.99)	0.19
21	Use of mosquito coils	383	2.38 (1.46–3.88)	*p < 0.001*	1.95 (0.95–4.03)	0.07
22	Daily light incense sticks	383	0.91 (0.56–1.43)	0.65	0.72 (0.37–1.39)	0.33
23	Place of cooking combined with living area	383	2.25 (1.42–3.69)	*p = 0.001*	2.75 (1.09–6.95)	*0.03*
24	Unclean fuel usage	378	1.69 (1.06–2.71)	*0.027*	1.11 (0.56–2.18)	0.77
25	Unsatisfactory ventilation in kitchen	370	1.60 (1.002–2.572)	*0.050*	1.25 (0.67–1.94)	0.16
26	Father ever smoked	383	1.64 (1.02–2.62)	*0.038*	1.09 (0.55–2.15)	0.80
27	Higher exposure to vehicle emissions	383	1.19 (0.75–1.90)	0.46	1.17 (0.60–2.27)	0.64
28	Having work place/industry	383	1.87(1.16–3.01)	*0.010*	1.27 (0.66–2.45)	0.47

Note: *OR Odds ratio

**CI Confidence Intervals

Significant *p* values are in italic

Maternal (*p* < 0.001) and paternal (*p* < 0.001) histories of wheezing, playing with soft toys in the sleeping area (*p* = 0.004), place of cooking combined with the living area (*p* = 0.03) and unsatisfactory ventilation in the sleeping area (*p* < 0.001) were found to be significantly associated with increased ‘current wheezing’ through multivariate analysis in this study. Use of formula milk before six months of age (*p* = 0.014) was found to be protective through multivariate analysis.

In the Omnibus tests of coefficients, the Chi square value was 163.73 and was highly significant (*p* < 0.001) indicating that the data fit the model adequately. The Cox and Snell R square was 0.348 and the Nagelkerke’s R square was 0.512 indicating that the final model explained 34.8%–51.2% of the variance in ‘current wheezing’.

## Discussion

As far as we know this is the first study on prevalence of wheezing among preschool children in any study setting of Sri Lanka.

The study findings confirmed that residents of the USS in CMC were of lower levels of education and income. The sex distribution of children aged three to five years is very similar to the sex distribution of children under five years at national and district levels [17]. The percentage of fathers of the children included who had not received schooling in this study was higher (5.9%) than the national value of 3.8% [17]. The median family monthly income was Rupees 28,000 (USD 193.1) with an interquartile range of Rupees 20,000–35,000 (USD 137.9–241.3), which is lower than the national median household income of Rupees 30,814 (USD 212.5) according to the latest Household Income and Expenditure Survey in Sri Lanka in 2013 [27]. The findings of education level of parents indicated that it was far below in the USS compared to the rest of the country. Nearly 59% of the mothers and 55% of the fathers had been educated only up to grades nine to eleven (biological age equivalent of children in grades 9–11 at school is 14–16 years) at school. The proportions of fathers and mothers of the children in the study who had not received schooling were 5.9% and 2.6% respectively. The percentage of fathers who had not received schooling in this study was higher than the national value of 3.8% computed for both sexes. Though unemployment rate of 1.5% observed in the study among fathers was lower than the national value of 5.7%, the occupations were mostly in the categories of unskilled and daily wage earners [17].

Although most asthma and wheezing related studies among 6–7 and 13–14 year olds have followed the International Study of Asthma and Allergies in Childhood (ISAAC) methodology, the present study did not use the methodology as the focus was younger preschool aged children. Though some researchers have used a modified version of the ISAAC questionnaire adapted to the preschool age and study setting [28], its validity is not well researched. Furthermore, the present study did not consider preschool wheezing as the same entity as asthma as there is lack of chronicity which is expected in asthma [6]. Moreover, inflammatory reactions in preschool aged children have been shown to be different from those observed in older children with diagnosed asthma [29]. The problem of parental report of wheezing, leading to over or underestimation was overcome in the present study by documenting physician’s diagnosis indicative of wheezing illness. The fact that 73% of the those identified as having wheezing illness had documented evidence of a diagnosis in the present study indicate the validity of the estimations made.

This study only recruited children with CHDRs noting that in Sri Lanka there is a very high availability rate of CHDRS (98.61%) [30]. However, this fact precludes generalizing the results of the present study to all children of this age group.

The findings showed that prevalence rates of ‘ever wheezing illness’ and ‘current wheezing illness’ of the present study to be higher than the previous estimations of asthma in older school children in the same study setting and in other settings in Sri Lanka (Table 4). Differences in the age groups of the children and the methodologies even among the studies that had been conducted in the CMC preclude the use of the findings to suggest recent increases of prevalence of wheezing illnesses.

**Table 4:** Comparison of prevalence estimates between the present study and studies conducted on asthma in older school children

Study	Area of study	Age group (years)	‘Ever wheezing’ (95% CI**)	‘Current wheezing’ (Within 12 months)
Present study	USS, Colombo	3–5	38% (33.6–42.5%)	21.3% (17.6–25.0%)
Samarasinghe, 2007	Colombo, CMC*	5–11	22.7% (NM***)	12.8% (NM)
Danansuriya, 2009	Gampaha	12–14	19.4% (17.3–21.3%)	16.7% (14.8–18.6%)
Nandasena et al.,2012	Colombo CMC Panadura	7–10	NM (NM)	20.8% (NM)

Note:*CMC Colombo Municipal Council

**CI Confidence Intervals

***NM- Not mentioned

USS Under-served settlements

The prevalence of ‘ever wheezing’ of the present study was also higher than the global prevalence of 34% among preschool aged children. There may be several reasons as to the high estimations. Among the preschool children the present study only included the older categories of three to five years. Globally, the prevalence of asthma and wheezing illnesses is higher in the younger children than the older children [2]. Higher estimations could also be attributed to the study setting being urban and underserved. High levels of wheezing illnesses are usually observed in underserved communities than in rural, semi urban and more affluent urban communities [31, 32]. In USSs, high prevalence has been attributed to exposure to unhealthy environment with poor ventilation and overcrowding [24].

The assessment of associations was carried out only for ‘current wheezing illness’ occurring in the 12 months period prior to the study for several reasons. Of those who had a history of wheezing occurring more than one year ago, a majority had had their wheezing in infancy. Wheezing in infancy is purported to have causal and risk factors which are different [26, 33] to the associated factors of wheezing illnesses in late preschool children. The assessment of factors associated in the preceding 12 months gives those which are related more recently to the wheezing illness and hence, useful in preventing exacerbations. Therefore, this information will be useful for healthcare workers of the area in identifying factors that can be modified to avoid exacerbations. In addition, the period of recall is also more likely to be valid and reliable. As the scope of this study was to assess the factors associated with ‘current wheezing illness’ the analysis was confined to those who had wheezing occurring within the 12 months period prior to the study.

In the present study, introduction of formula milk before six months of age was found to be protective through both bivariate and multivariate analyses. Not being exclusively breast fed was found to increase the risk of ‘current wheezing’ illness in multivariate analysis. However, it was not a significant factor (*p* = 0.06). Research articles on wheezing and asthma among older school children, preschool children and infants demonstrate varying results with regard to formula feeding and breast feeding practices. Interestingly, non-exclusive breast feeding practices and formula feeding both have been found to be protective in infants [34]. It is postulated that early introduction of cow’s milk can modify immunological reactions and hypersensitivity states [35]. Formula milk introduction before three months of age leading to increased risk of wheezing among preschool children has been reported in literature [36]. Exclusive breast feeding only for the first four months of life [34] or more [33] are also known to be protective in both preschool children and infants respectively.

Associated with ‘current wheezing’ illnesses among children of three-five years in the present study indicated that male sex (*p* = 0.056) was not significantly associated with an increased risk of ‘current wheezing’ when adjusted for the confounding effects of other factors. However, increased risk of male children in developing wheezing illnesses is well established in research [15, 19, 22, 37–40]. The present study identified both maternal (*p* < 0.001) and paternal (*p* < 0.001) histories of wheezing or asthma were significantly associated with ‘current wheezing illness’ of children in multivariate analyses. Similar results have been shown in many studies conducted in the same age category in other countries [35] and among older school children [21, 22, 41].

The present study revealed several factors related to the indoor and outdoor environment to be associated with ‘current wheezing’ through multivariate analysis.

Place of cooking combined with living area (*p* = 0.03) was found to be significantly associated with ‘current wheezing’ in this study. Furthermore, observation of houses revealed that 91% of households did not have a chimney in the area of cooking. Through bivariate analysis unsatisfactory ventilation in kitchen area was found to be significantly associated with ‘current wheezing’. Pollutants from incomplete cooking fuel combustion are known to increase wheezing illness in children under five years [21, 42]. Though LPG is not an entirely ‘clean’ fuel source it was considered as clean, compared to the other two types used in the study setting. In the present study, use of ‘unclean’ type of fuel such as kerosene and firewood, was significantly associated with ‘current wheezing illness’ (*p* = 0.027) through bivariate analysis. Time activity studies conducted by Klepeis and others in 2001 show that younger children can spend up to 90% of their time indoors [42]. Thus, the place of cooking combined with the living area, along with lack of chimney can contribute to the development or triggering of wheezing among younger children who spend a greater part of their time indoors. Bed nets as well as soft toys can trap dust particles and thus, trigger wheezing. In the multivariate analysis, playing with soft toys (*p* = 0.004) and unsatisfactory ventilation in the sleeping area (*p* < 0.001) were found to be significantly associated with ‘current wheezing’ as well. This also indicates that the sleeping areas of children in these under-served settlements also need to be modified suitably as well.

Having a father who had ever smoked in the house was identified as significantly associated with ‘current wheezing illness’ in this study (*p* = 0.038) in the bivariate analysis, similar to other studies done among children under five years [15, 43]. Though maternal smoking has been identified as increasing the risk of wheezing in children of all ages [15], Sri Lankan studies of the older child [19, 20] have not identified this as a factor. There were no mothers who smoked in the present study thus an association could not be assessed.

Living near a place of work/activities/industry generating emissions located within 150 m of the household, was found to be statistically significant (*p* = 0.010) only by bivariate analysis. This is in agreement with other studies where being resident in urban areas [21] and living near industrial areas [44] have been identified as important factors.

The main vehicle type observed on by-roads of underserved settlements was three wheelers. Though the timing is infrequent, the emissions of the three-wheelers release significant amounts of pollutants to the air as the fuel emission standards and emission reducing technologies have not been fully incorporated into three wheelers as they have been for passenger cars [45]. Hence combining ‘infrequent traffic’ with ‘heavy traffic’ as the exposed category for fuel emissions was carried out. Through both bivariate and multivariate analyses higher exposure to vehicle emissions was not significantly associated with ‘current wheeze’.

There are several limitations in this study. The use of a cross-sectional design did not allow exploration of the temporal relationship of the factors and the outcome. As diet is a complex factor with difficulties in measuring, this was not be evaluated as it was considered beyond the scope of this study.

## Conclusions

The magnitude of wheezing illnesses among three to five year old children resident in low-income settlements of an urban area was found to be high, needing the attention of healthcare authorities. Children with a history of maternal and/or paternal wheezing should be targeted for early interventions to prevent exacerbations of wheezing illnesses. Interventions should also focus on the indoor environmental factors that were found to be associated with wheezing illnesses, in order to avoid exacerbations.

## Abbreviations

CHDR: Child Health Development Record
CMC: Colombo Municipal Council
ISAAC: International Study of Asthma and Allergies in Childhood
LPG: Liquid Petroleum Gas
USS: Under-served settlements
